# Below the Aortic Annulus: Multiphase CT Features of an Interventricular Membranous Septal Aneurysm

**DOI:** 10.3390/diagnostics16182894

**Published:** 2026-09-08

**Authors:** Paweł Gać, Marcin Witkowski, Rafał Poręba

**Affiliations:** 1 Department of Environmental Health, Occupational Medicine and Epidemiology, Wroclaw Medical University, 50-345 Wroclaw, Poland; 2 Centre for Diagnostic Imaging, 4th Military Hospital, 50-981 Wroclaw, Poland; 3 Department of Biological Principles of Physical Activity, Wroclaw University of Health and Sport Sciences, 51-612 Wroclaw, Poland

**Keywords:** coronary artery bypass grafting, coronary computed tomography angiography, left ventricular outflow tract, interventricular membranous septal aneurysm

## Abstract

We present interesting diagnostic images from a 77-year-old man with chronic coronary artery disease, a history of coronary artery bypass grafting (CABG), and no documented history of myocardial infarction who underwent coronary computed tomography angiography (CCTA) to assess graft patency. No operative report, previous medical documentation, or earlier cardiac imaging studies were available. CCTA demonstrated patent grafts from the left internal mammary artery to the left circumflex coronary artery (LIMA–LCx) and from the ascending aorta to a diagonal branch (aorto-diagonal graft). A stump-like protrusion of the ascending aorta was considered most consistent with the residual proximal segment of an occluded saphenous vein graft, presumably previously directed to the right coronary artery. In addition, an incidental broad-necked, partially lobulated, contrast-filled outpouching measuring approximately 28 × 17 × 29 mm was identified within the basal membranous interventricular septum. Multiplanar and multiphase reconstructions demonstrated a subannular neck communicating directly with the left ventricular outflow tract (LVOT), preservation of the muscular interventricular septum, separation from the sinus of Valsalva, and phase-dependent rightward protrusion toward the basal right ventricle. These anatomical features favored an interventricular membranous septal aneurysm (IVMSA). Because no CMR was performed, remote clinically silent ischemic injury could not be completely excluded. Overall, the lesion was considered most consistent with IVMSA; its congenital or developmental origin and temporal evolution could not be established in the absence of prior imaging and clinical documentation. This case highlights the diagnostic value of thin-section, multiphase CCTA for characterizing a septal outpouching and distinguishing IVMSA morphology from a sinus of Valsalva aneurysm and acquired postoperative or ischemic alternatives.

**Figure 1:**
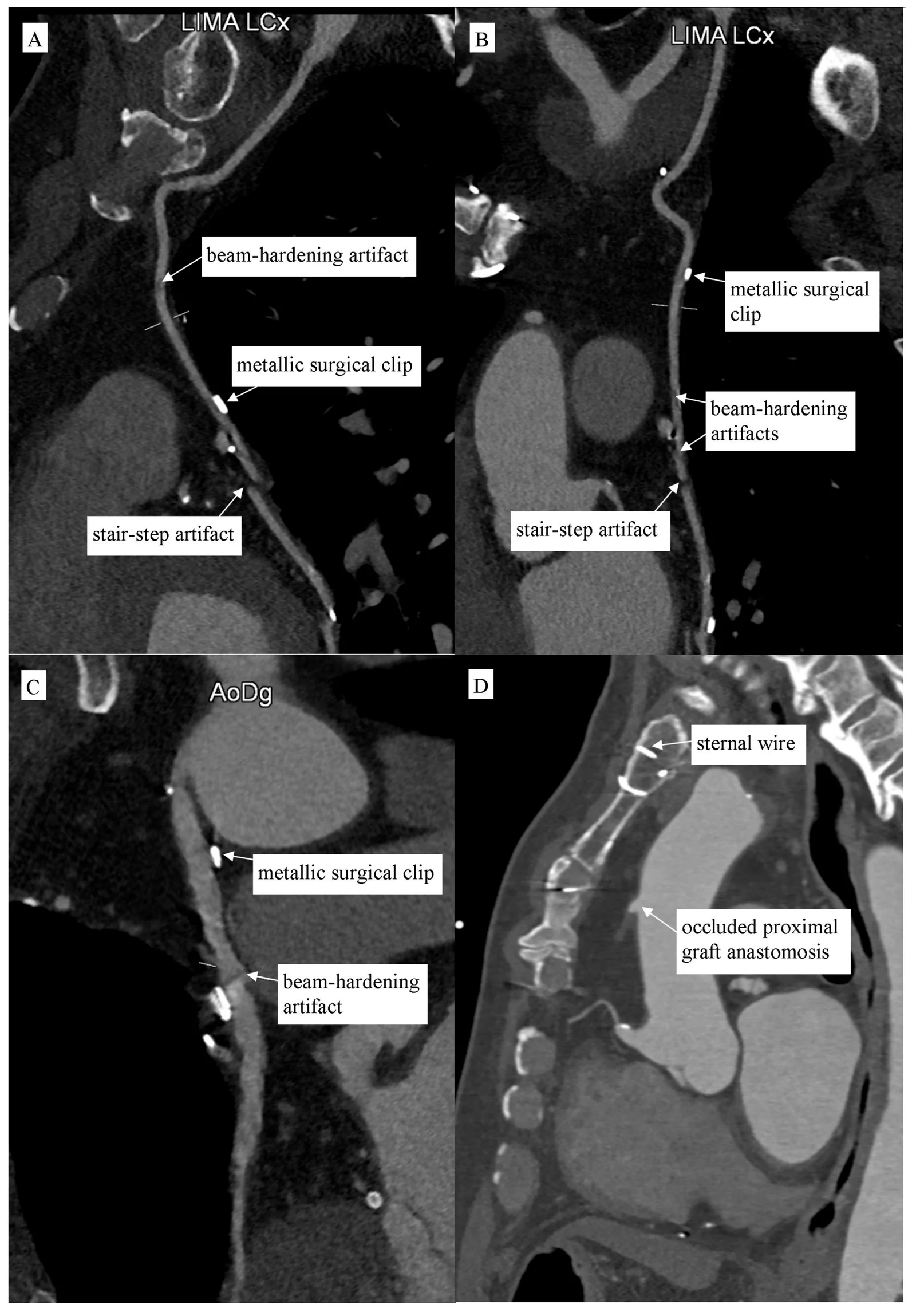

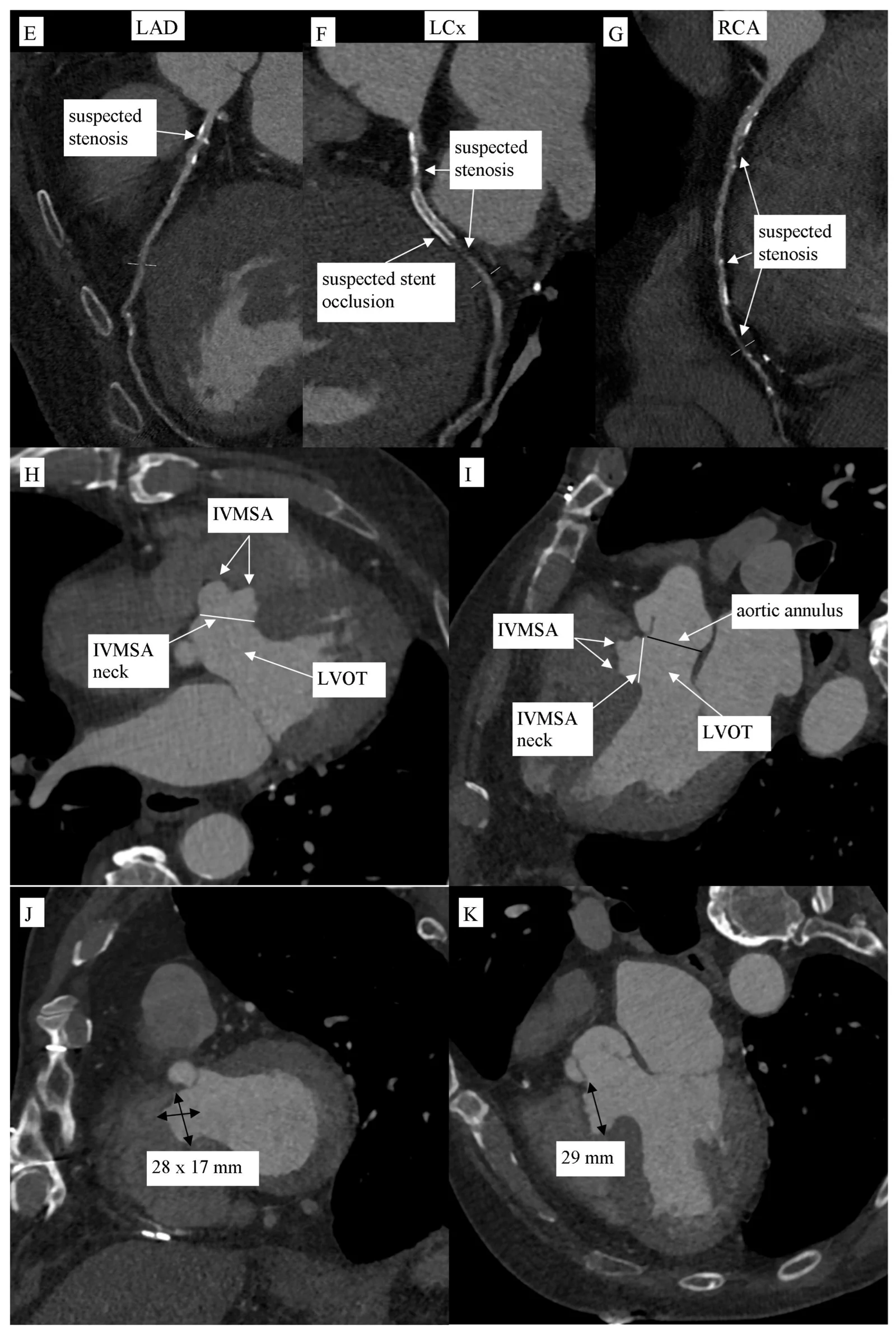

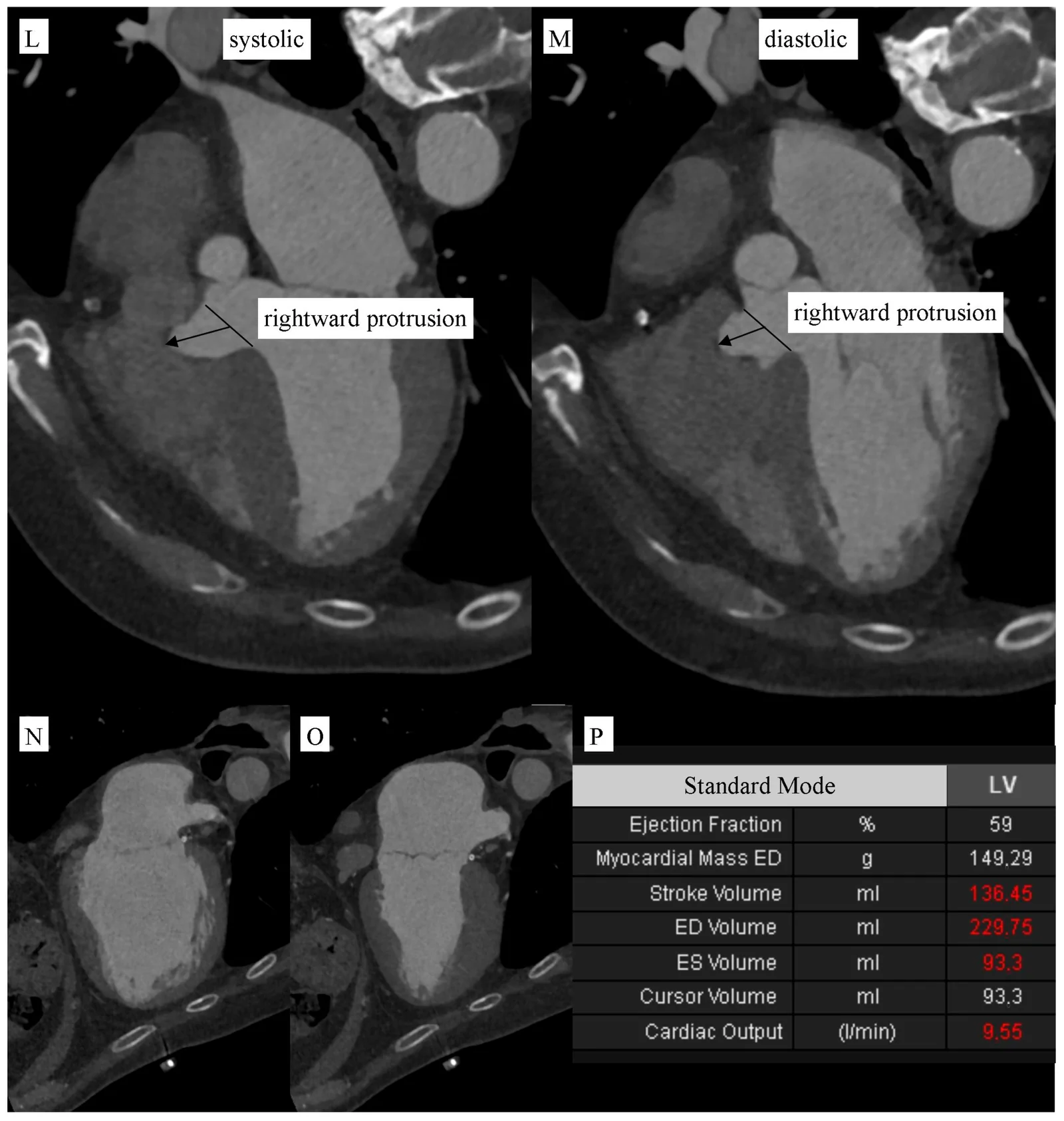
A 77-year-old man with chronic coronary artery disease, a history of coronary artery bypass grafting (CABG), and no documented history of myocardial infarction was referred for coronary computed tomography angiography (CCTA) to assess bypass graft patency. The septal outpouching described below was an incidental finding and was not the indication for CCTA; based on the available documentation, no symptom could be specifically attributed to the lesion. No previous medical records, operative reports, or prior cardiac imaging studies were available for review. CCTA was performed using a third-generation dual-source CT scanner (SOMATOM Force; Siemens Healthineers, Erlangen, Germany). Following the intravenous administration of 65 mL of iomeprol at a concentration of 400 mg I/mL and a flow rate of 5.0 mL/s, retrospective ECG-gated acquisition with ECG-based radiation dose modulation was performed at 100 kV and a heart rate of approximately 70 beats/min. Images were reconstructed at a slice thickness of 0.6 mm. CCTA demonstrated patent LIMA–LCx (panels (**A**,**B**)—CPR reconstruction of the LIMA–LCx bypass graft in two views rotated by 90°) and aorto-diagonal bypass grafts (panel (**C**)—CPR reconstruction of the Ao–Dg bypass graft), with preserved contrast opacification throughout their evaluable courses, including the distal anastomoses. A focal, contrast-filled, stump-like outpouching of the tubular ascending aorta was identified at the presumed site of a proximal venous graft anastomosis (panel (**D**)—sagittal MPR reconstruction). As no contrast-opacified continuation of the graft was visualized, this finding was considered most consistent with the residual proximal stump of an occluded saphenous vein graft, most likely previously directed to the right coronary artery. Native coronary arteries showed diffuse, predominantly calcified atherosclerotic plaques (panels (**E**–**G**)—CPR reconstructions of the LAD, LCx, and RCA, respectively). Extensive calcification and associated blooming artifacts precluded reliable grading of luminal stenosis severity. A stent in the left circumflex coronary artery showed no convincing intraluminal contrast opacification, suggesting stent occlusion; however, detailed assessment was limited by stent-related artifacts (panel (**F**)). In addition to the coronary and bypass graft findings, CCTA revealed an incidental, broad-necked, irregular, partially lobulated contrast-filled outpouching arising from the basal membranous interventricular septum (panel (**H**)—axial view). The lesion communicated directly with the left ventricular outflow tract and projected rightward toward the basal right ventricle and right ventricular outflow tract. The neck of the lesion remained below the aortic annulus, with no evidence of direct communication with the sinus of Valsalva (panel (**I**)—MPR reconstruction, LVOT view). The broad aneurysmal neck is shown in panels H and I. The broad aneurysmal neck measured approximately 28 × 19 mm in end-diastole and 26 × 19 mm in end-systole. The lesion measured approximately 28 × 17 mm in short-axis reformations and up to 29 mm in the three-chamber long-axis view (panel (**J**)—MPR reconstruction, short-axis view, panel (**K**)—MPR reconstruction, three-chamber view). Comparison of end-systolic and end-diastolic reconstructions demonstrated a phase-dependent change in its configuration, with greater rightward bulging of the IVMSA during systole (panel (**L**)—end-systolic MPR reconstruction, panel (**M**)—end-diastolic MPR reconstruction). No definite intraluminal thrombus or disruption of the muscular interventricular septum was identified. Taken together, the subannular origin, direct continuity with the LVOT, preserved muscular septum, separation from the sinus of Valsalva, and phase-dependent rightward protrusion were most consistent with IVMSA. The left ventricle was enlarged, with preserved global systolic function (ejection fraction, 59%) (panel (**N**)—end-diastolic MPR reconstruction, two-chamber long-axis view, panel (**O**)—end-systolic MPR reconstruction, two-chamber long-axis view, panel (**P**)—left ventricular functional parameters). The aortic valve was tricuspid, without visible valvular calcification. **Commentary:** An interventricular membranous septal aneurysm (IVMSA) is a localized, thin-walled outpouching arising from the small fibrous component of the interventricular septum. This portion is located at the cardiac base, immediately below the aortic annulus, near the junction of the right and non-coronary aortic cusps. Because the membranous septum lacks contractile myocardium and is exposed to the systolic pressure gradient between the left and right ventricles, aneurysmal tissue typically protrudes rightward into the basal right ventricle and may extend toward the right ventricular outflow tract (RVOT) [1,2,3]. In the present case, the subannular origin, direct continuity with the left ventricular outflow tract, and rightward phase-dependent change in configuration were characteristic of this anatomical location. IVMSA may occur as an isolated anomaly but is strongly associated with perimembranous ventricular septal defects (VSDs) [1,3,4]. A commonly proposed mechanism involves aneurysmal transformation of tissue participating in the partial or complete spontaneous closure of a perimembranous VSD. Adherence of tricuspid valve tissue, accessory endocardial cushion tissue, or organized fibrous deposits may progressively restrict the defect, while continued exposure of the sealing tissue to left ventricular systolic pressure may result in the formation of a thin-walled aneurysmal sac [2,3,4]. This developmental mechanism is well described in the literature; however, in the present patient neither a congenital/developmental origin nor the time of lesion formation can be established because no prior imaging, operative report, or relevant historical documentation was available. The principal diagnostic challenge is differentiation from an aneurysm of the sinus of Valsalva and from an acquired post-infarction or postoperative septal pseudoaneurysm. A sinus of Valsalva aneurysm originates above the aortic annulus and communicates directly with an aortic sinus, whereas an IVMSA arises below the annulus and communicates with the left ventricular outflow tract [1,2,3]. Multiplanar reconstructions aligned with the aortic root and LVOT are therefore more informative than the shape of the outpouching alone. In contrast, an acquired post-infarction septal aneurysm or pseudoaneurysm would be expected to arise in relation to injured muscular septum, with thinning or disruption of myocardium and, when tissue characterization is available, evidence of adjacent ischemic scar. In this patient, the subannular origin, direct LVOT continuity, preserved muscular interventricular septum, intact aortic root contour, and phase-dependent rightward protrusion favored IVMSA. The absence of a documented myocardial infarction was considered only supportive and not exclusionary, because remote clinically silent ischemic injury is possible in an elderly patient with established coronary artery disease. CMR was not performed and therefore myocardial scar could not be definitively excluded. The previous CABG and the separate stump-like outpouching of the ascending aorta constituted an important diagnostic distraction rather than a plausible anatomical source of the septal lesion. Once IVMSA is recognized, imaging assessment should extend beyond confirming its origin. The report should include the maximum dimensions and width of the aneurysmal neck, its relationship to the aortic annulus, tricuspid valve, and RVOT, and any phase-dependent change in morphology. Color Doppler echocardiography is important for determining whether a residual left-to-right shunt persists across an associated perimembranous VSD [2,3,4]. The aneurysmal sac should also be assessed for thrombus because blood stasis within the cavity may predispose to systemic thromboembolism and cerebral infarction [1,5,6]. Larger lesions may produce dynamic or fixed RVOT obstruction [2,7] or distort an adjacent aortic cusp, resulting in cusp prolapse and progressive aortic regurgitation [3]. Finally, because the atrioventricular bundle and proximal bundle branches pass close to the posterior and inferior margins of the membranous septum, electrocardiographic assessment for atrioventricular block, bundle-branch block, or ventricular arrhythmia should complement the imaging findings [2,3]. In the present case, no definite intracavitary thrombus, direct communication with the sinus of Valsalva, or muscular septal disruption was identified on CCTA; however, echocardiographic evaluation of residual shunting, RVOT hemodynamics, and aortic valve function remains appropriate. This report has several limitations. First, no previous medical records, operative report, or earlier cardiac imaging studies were available; therefore, the onset and temporal evolution of the septal lesion and the precise details of the CABG procedure could not be established. Second, the diagnosis was based primarily on multiphase CCTA morphology and should therefore be regarded as most consistent with IVMSA rather than histopathologically proven. Third, echocardiographic data were not available to determine whether a residual perimembranous VSD or left-to-right shunt was present, to quantify any RVOT gradient, or to assess subtle aortic or tricuspid valve dysfunction. CMR was also not performed and could have provided additional tissue characterization, particularly for detection of myocardial scar and a small mural thrombus. The lesion was incidental, and the available documentation did not permit attribution of specific symptoms to it. Dedicated clinical or imaging follow-up of the septal lesion was not available. Finally, extensive coronary calcification and stent-related artifacts limited reliable evaluation of the native coronary arteries, although they did not materially affect anatomical assessment of the septal outpouching. In summary, this case illustrates that not every cardiac outpouching identified in a patient after CABG should be assumed to be postoperative. The decisive diagnostic features were anatomical: the neck arose below the aortic annulus, communicated with the LVOT, remained separate from the sinus of Valsalva, and was associated with an intact muscular interventricular septum and phase-dependent rightward protrusion. These findings favored IVMSA over a sinus of Valsalva aneurysm and an acquired septal pseudoaneurysm. Nevertheless, because prior imaging and CMR were unavailable, the developmental origin and timing of the lesion cannot be established and remote silent ischemic injury cannot be completely excluded. Accordingly, thin-section multiplanar and multiphase CCTA can provide a critical anatomical roadmap for identifying a lesion most consistent with IVMSA when clinical history is incomplete, while complementary echocardiography and, where clinically indicated, CMR may address residual shunting, hemodynamic consequences, and tissue characterization.

## Data Availability

The original contributions presented in this study are included in the article. Further inquiries can be directed to the corresponding author.

## Abbreviations

The following abbreviations are used in this manuscript:

Ao	Aorta
CABG	Coronary artery bypass grafting
CCTA	Coronary computed tomography angiography
CPR	Curved planar reconstruction
CT	Computed tomography
Dg	Diagonal branch
ED	End-diastolic
ES	End-systolic
IVMSA	Interventricular membranous septal aneurysm
LAD	Left anterior descending artery
LCx	Left circumflex artery
LIMA	Left internal mammary artery
LV	Left ventricle
LVOT	Left ventricular outflow tract
MPR	Multiplanar reconstruction
OM	Obtuse marginal branch
RCA	Right coronary artery
RVOT	Right ventricular outflow tract
VSD	Ventricular septal defect
